# Supplementary figures and images for: Plasmodium falciparum stomatin-like protein forms a putative complex with a metalloprotease in distinct mitochondrial loci
Source: PLoS Pathog. 2026 Feb 9;22(2):e1013922. doi: 10.1371/journal.ppat.1013922 (PMC12912694; doi:10.1371/journal.ppat.1013922)

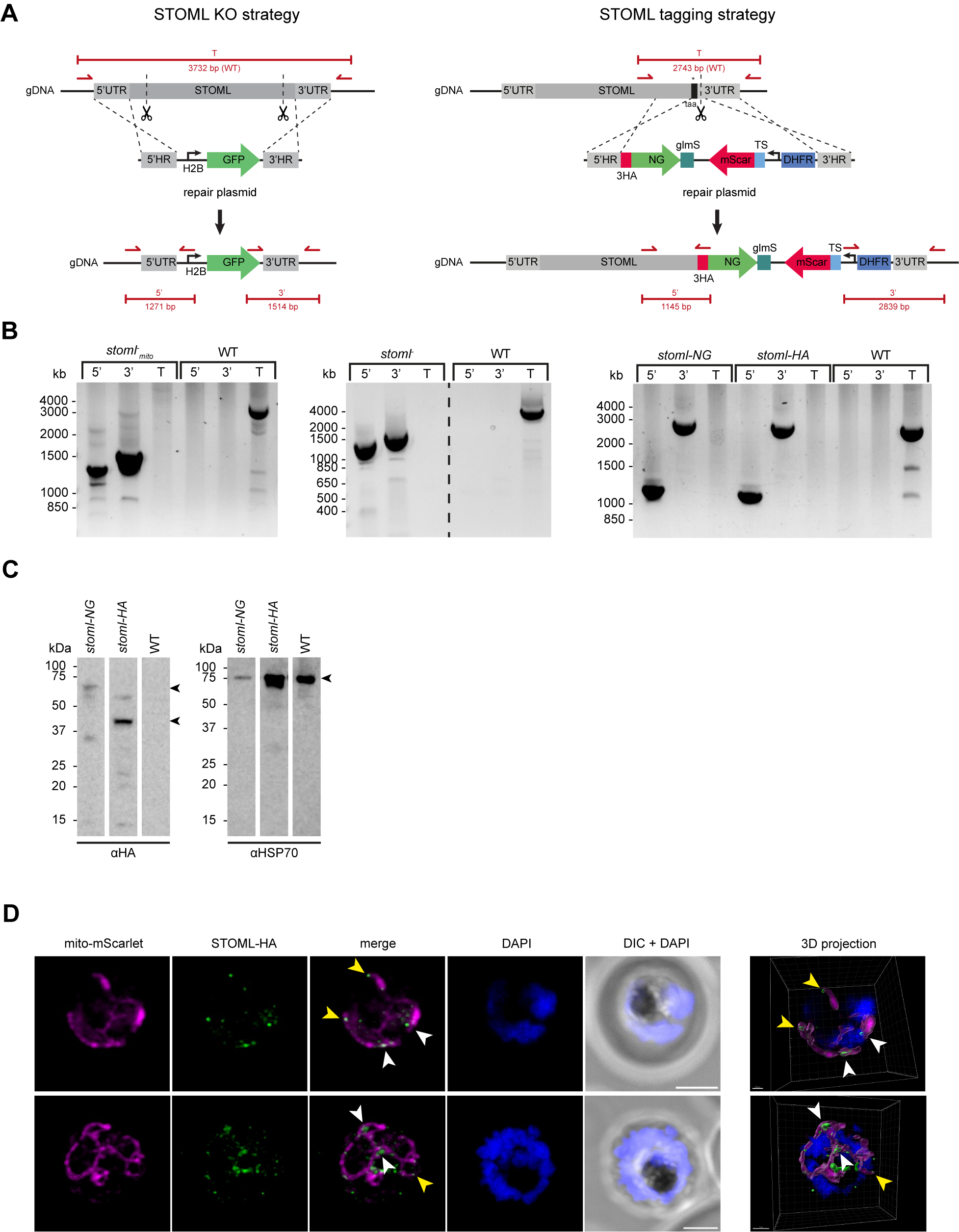

Supplement: S1 Fig — A) Schematic overview of STOML tagging and KO strategy. For tagging of STOML with 3HA-NG-glmS or 3HA-glmS, CRISPR-Cas9 (indicated by scissors) is used to introduce a double-strand break to facilitate integration of the linear repair constructs 3HA(-NG)-glmS tag directly after STOML before the stop codon, while at the same time integrating a mito-mScarlet mitochondrial marker and a DHFR drug selection cassette. For STOML KO, two CRISPR-Cas9 introduced DNA breaks at the 5’ and 3’ of the gene will be repaired by the linearized HDR plasmid. After integration, STOML will be replaced by GFP under the control of the H2B promotor. B) Diagnostic PCR of stoml-NG, stoml-HA, stoml- and stoml-(mito) parasite lines with integration specific primer combinations (indicated in panel A), demonstrating successful 5’ and 3’ integration and the absence of WT parasites (T = total). C) Western blot analysis showing expression of STOML-3HA-NG (73 kDa) and STOML-3HA (47 kDa) at expected sizes using anti-HA antibody and anti-HSP70 for loading control. D) Fluorescence microscopy of stoml-HA with anti-HA antibody (green), mito-mScarlet mitochondrial marker (magenta), DAPI for DNA visualization (blue), and DIC. Images are maximum intensity projections of Z-stack confocal Airyscan images, except images in right panel, which are 3D visualizations generated with Imaris analysis software. Arrowheads indicate PfSTOML-3HA signal at mitochondrial branching points (white) or mitochondrial branch endings (yellow). Scale bars are 2 µm for maximum intensity projections and 1 µm for 3D visualizations. (TIF) [file ppat.1013922.s001.tif]

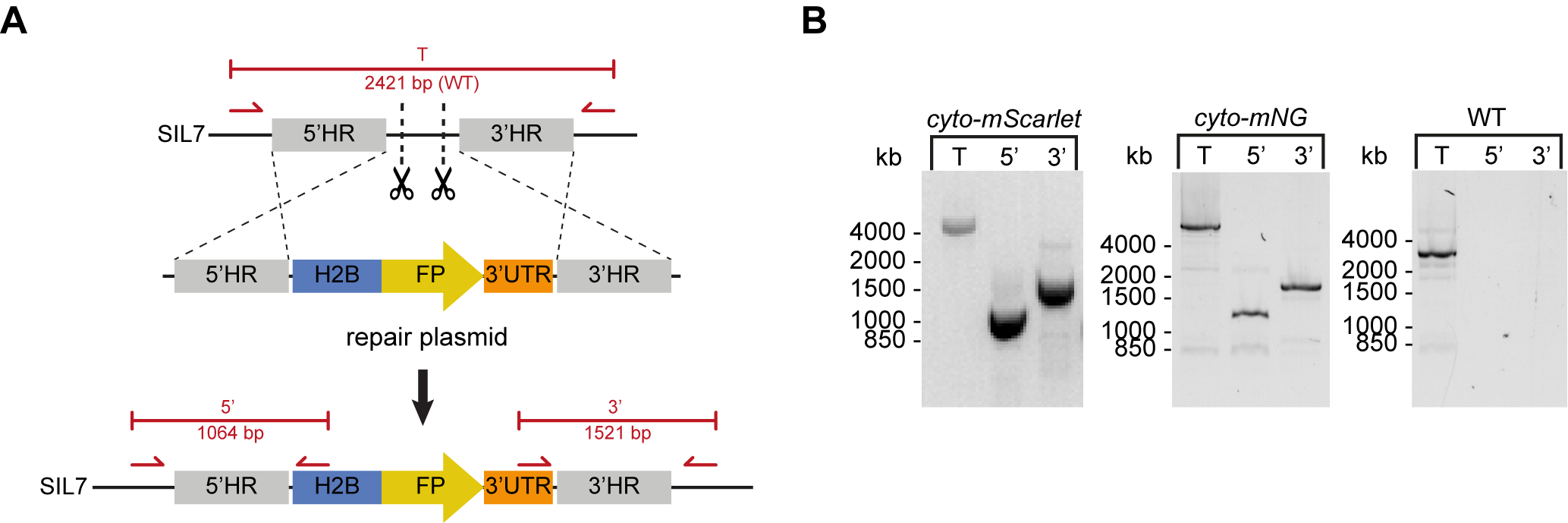

Supplement: S2 Fig — A) Schematic overview of transfection strategy to generate cyto-mScarlet and cyto-mNG. CRISPR-Cas9 and two guides were used to generate double stranded breaks in a silent intergenic locus (SIL7), characterized in Verhoef et al. [29] (indicated by scissors). DNA breaks are repaired by double homologous recombination with a repair plasmid containing 5’ and 3’ homology regions (HRs) and a fluorescent protein (FP, mScarlet or mNeonGreen) under the control of the H2B promotor and PBANKA_142660 bidirectional 3’UTR. B) Diagnostic PCR of cyto-mScarlet and cyto-mNG parasite lines with integration-specific primer combinations (indicated in panel A), demonstrating successful 5’ and 3’ integration and the absence of WT parasites (T = total). (TIF) [file ppat.1013922.s002.tif]

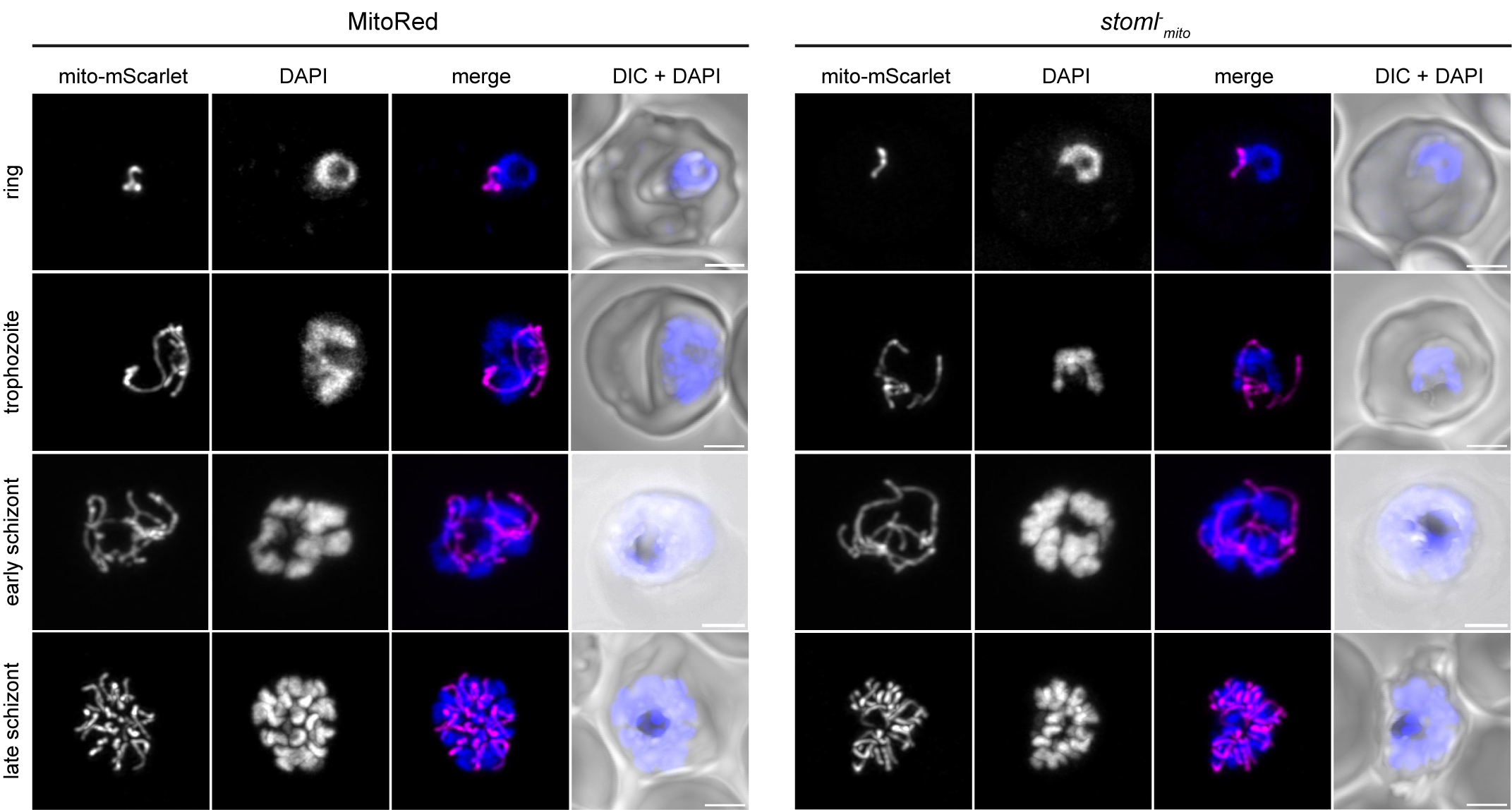

Supplement: S3 Fig — Fluorescent microscopy of stoml-mito and MitoRed (WT) parasites during ring, trophozoite, early and late schizont stages. The mito-mScarlet signal is preserved after fixation and can be observed without antibody staining. DNA was stained using DAPI. Images are maximum intensity projections of Z-stack confocal Airyscan images. Scale bars, 2 µm. (TIF) [file ppat.1013922.s003.tif]

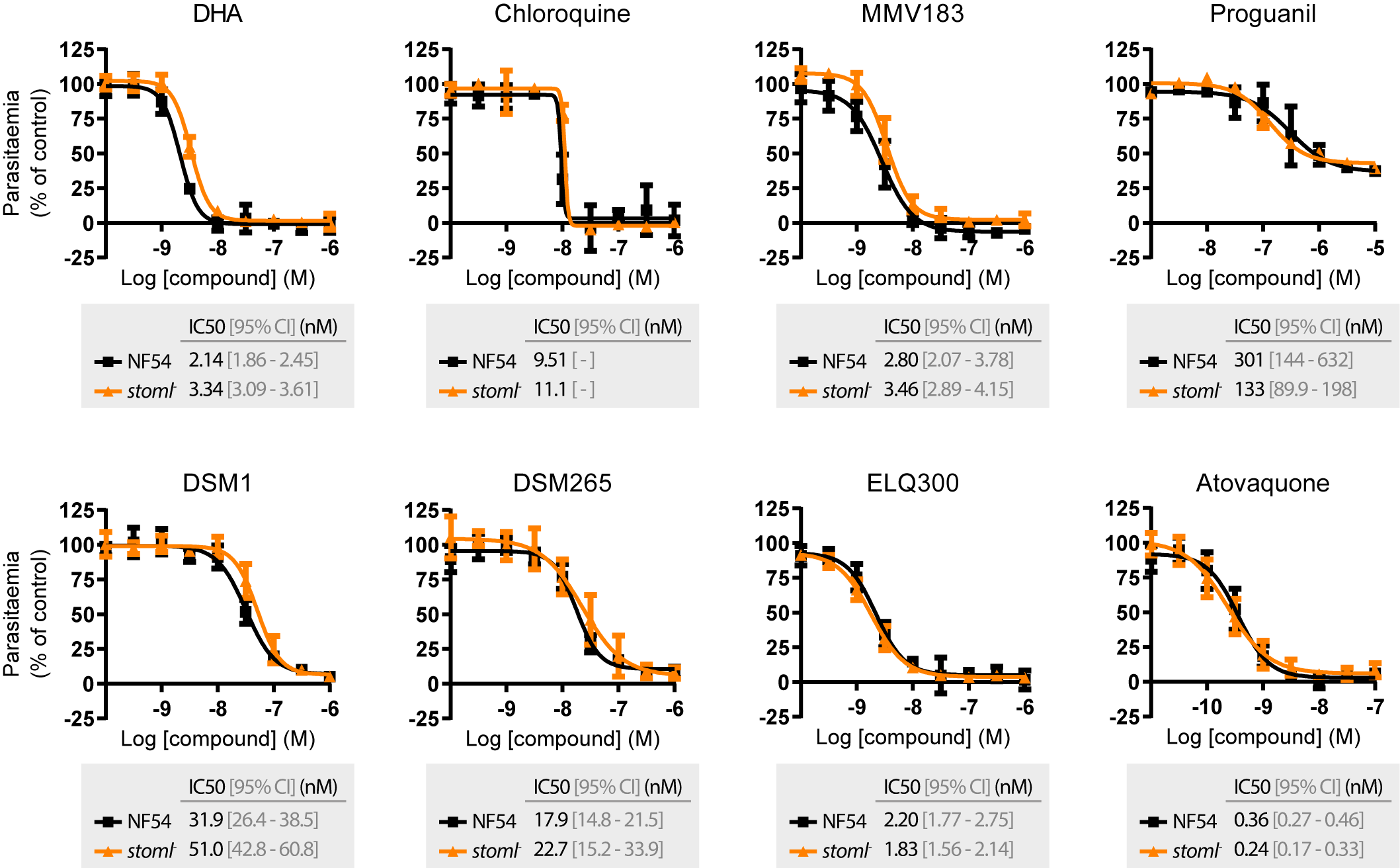

Supplement: S4 Fig — Drug sensitivity assay for P. falciparum NF54 and stoml- parasites. The graphs show average values for mean parasite density relative to controls for asexual blood-stage replication assay and represent one of the two independent replicates. Error bars indicate SEM determined from two technical replicates per experiment. The data were analyzed using nonlinear regression in GraphPad Prism. Proguanil, DSM1, DSM265, ELQ300, and Atovaquone are compounds targeting the parasite mitochondrion, while DHA, chloroquine, and MMV183 are non-mitochondrial compounds. (TIF) [file ppat.1013922.s004.tif]

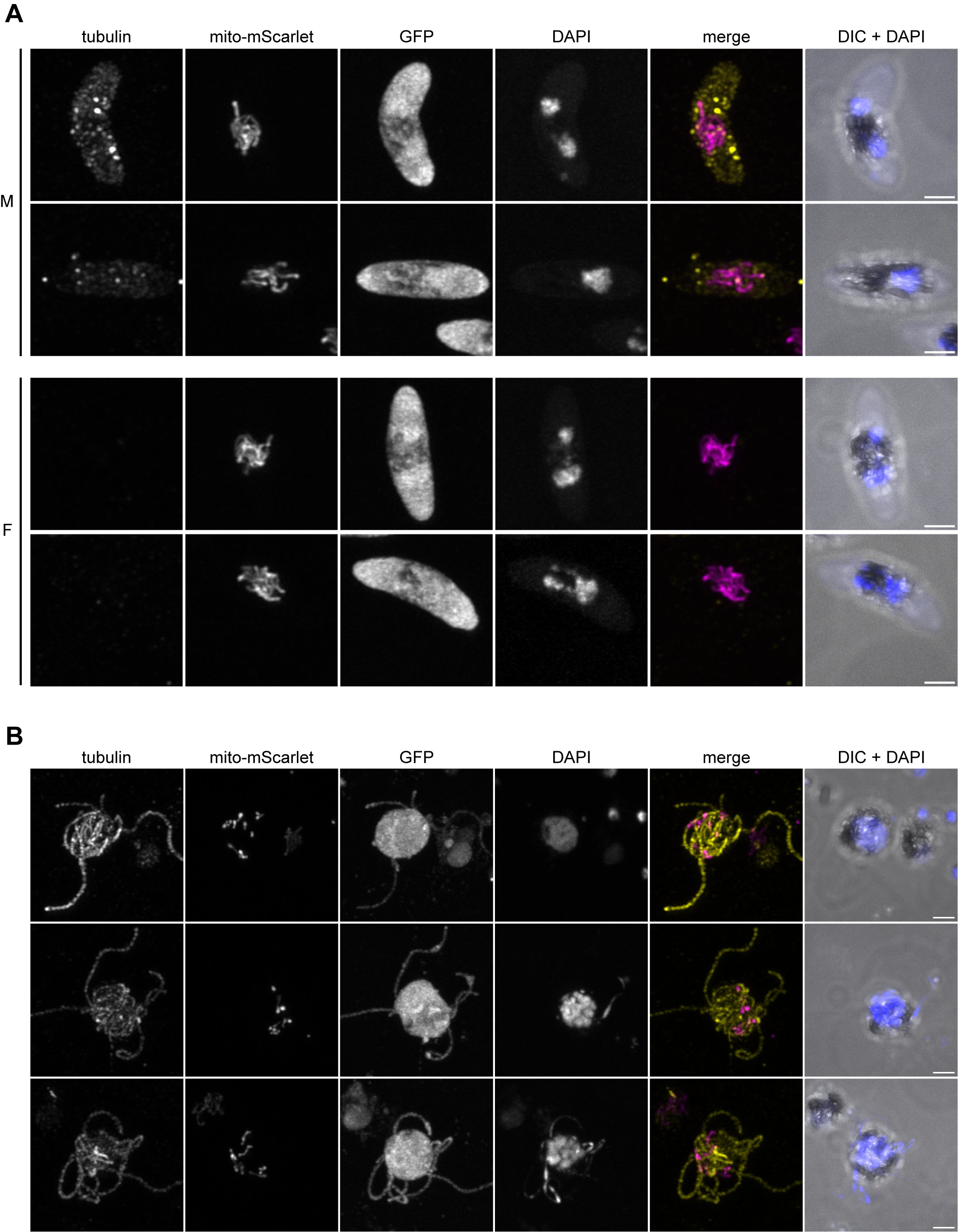

Supplement: S5 Fig — A) Fluorescent microscopy on male (M) and female (F) stoml-mito stage V gametocytes. Parasites were stained for tubulin (yellow) to distinguish male (high α-tubulin signal) from female (low α-tubulin signal) gametocytes. B) fluorescent microscopy on exflagellating stoml-mito male gametes at 20 minutes after activation. Parasites were stained with tubulin to visualize axonemes. A-B) Visualization of mito-mScarlet mitochondrial marker (magenta), cytosolic GFP, DAPI for DNA visualization (blue), and DIC. Images are maximum intensity projections of Z-stack confocal Airyscan images. Scale bars, 2 µm. (TIF) [file ppat.1013922.s005.tif]

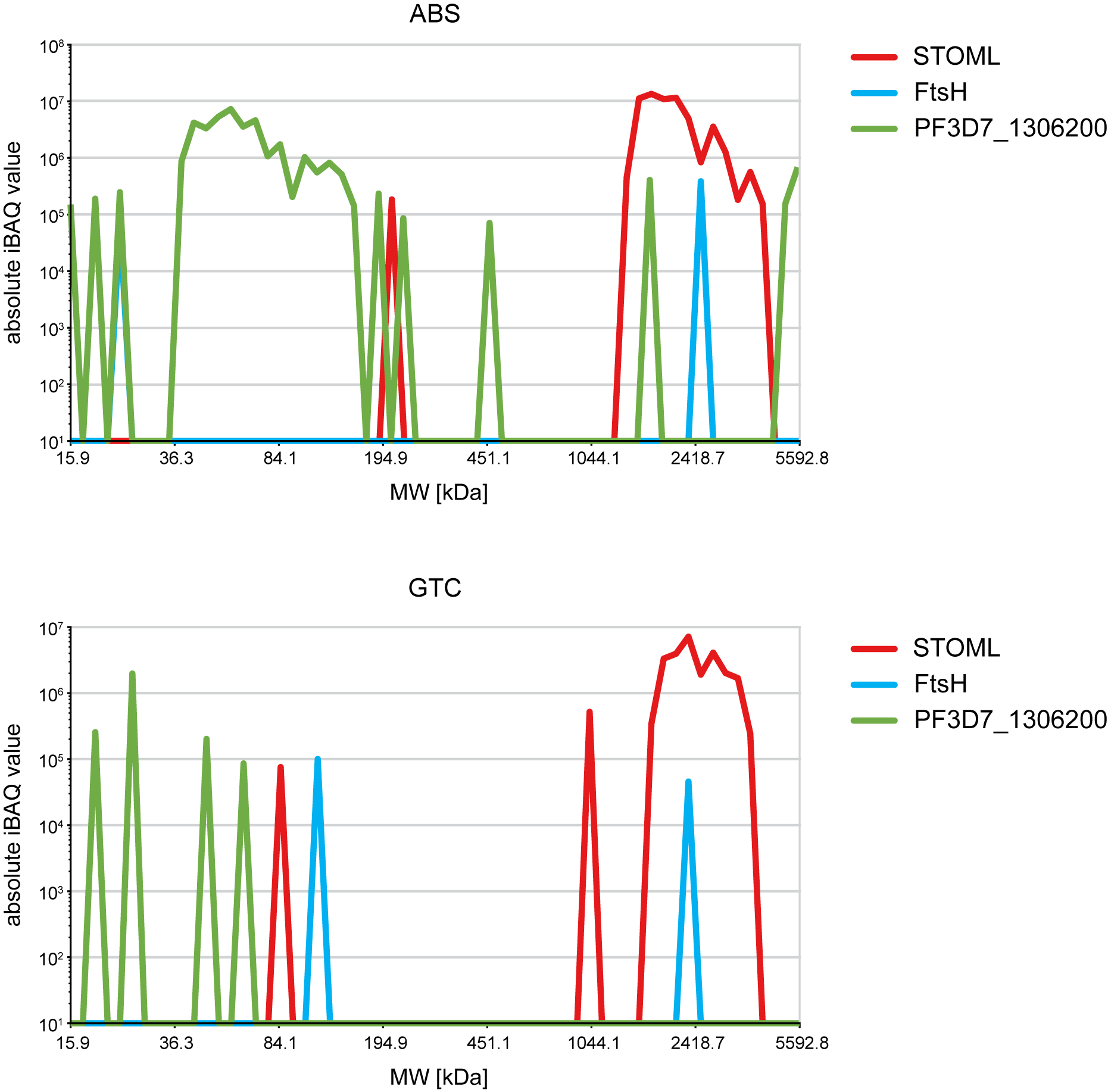

Supplement: S6 Fig — Line graphs based on previously published complexome profiling data [39] showing migration patterns and absolute protein abundance (iBAQ value, logarithmic scale) of STOML (red), FtsH (blue), and PF3D7_1306200 (green) in asexual blood stages (ABS) and gametocytes (GTC). Co-migration on blue native gel within the same molecular weight (MW) range (x-axis) indicates complex formation. (TIF) [file ppat.1013922.s006.tif]

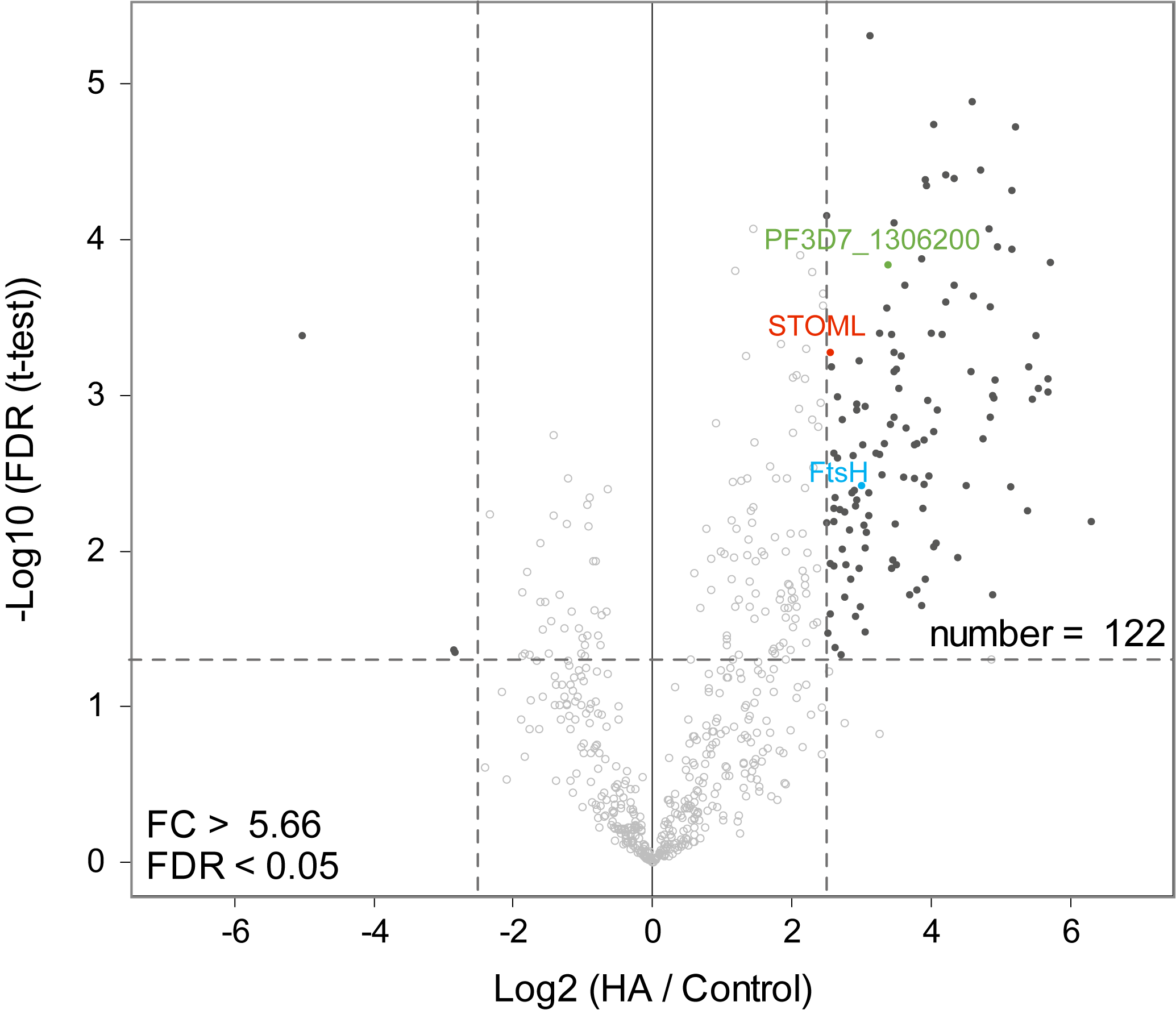

Supplement: S7 Fig — Anti-HA immunoprecipitation of PfSTOML-HA containing complexes. The volcano plot showing mean log2 fold changes (FC) and -log10 false discovery rate (FDR) for anti-HA pulldown in comparison with control pulldown. Horizontal and vertical dotted lines indicate log2 FC > 2.5 (FC > 5.66) and -log10 FDR > 1.301 (FDR > 0.05) respectively. Dark dots represent proteins that are highly enriched or reduced in the anti-HA pulldown compared to the control pulldown. (TIF) [file ppat.1013922.s007.tif]

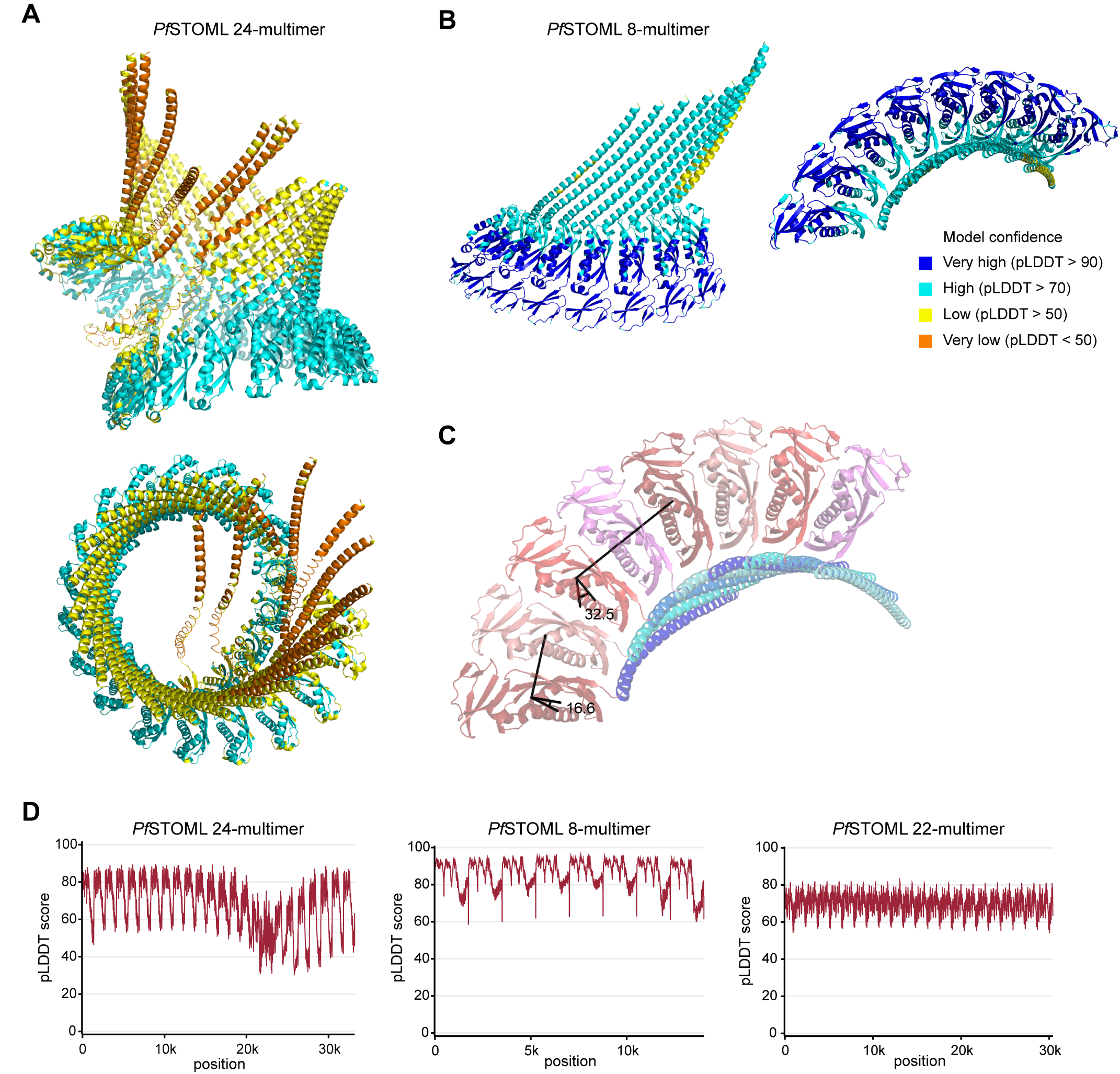

Supplement: S8 Fig — A) AlphaFold2 prediction of PfSTOML 24-multimer with side view (top) and top view (bottom). B) AlphaFold2 prediction of PfSTOML 8-multimer with side view (left) and top view (right). Coloring in A and B represent model confidence as indicated by the color legend in B. C) Top view of predicted PfSTOML 8-multimer structure, indicating the angles measured between SPFH domains of different STOML proteins in the complex. D) Graphs with pLDDT scores representing model confidence of predicted PfSTOML 24, 8, and 22 multimer structures. (TIF) [file ppat.1013922.s008.tif]
